# Nonalcoholic Steatohepatitis in a Patient with Ataxia-Telangiectasia

**DOI:** 10.1155/2014/761250

**Published:** 2014-01-06

**Authors:** Trinidad Caballero, Mercedes Caba-Molina, Javier Salmerón, Mercedes Gómez-Morales

**Affiliations:** ^1^Pathology Department, San Cecilio University Hospital and School of Medicine, University of Granada, Avenida de Madrid 11, 18012 Granada, Spain; ^2^Networked Biomedical Research Center for Hepatic and Digestive Diseases (CIBERehd), Carlos III Institute of Health, Spain

## Abstract

Ataxia-telangiectasia (A-T) is a rare disease characterized by neurodegenerative alterations, telangiectasia, primary immunodeficiency, extreme sensitivity to radiation, and susceptibility to neoplasms. A-T patients have inactivation of ataxia-telangiectasia-mutated (ATM) protein, which controls DNA double-strand break repair and is involved in oxidative stress response, among other functions; dysfunctional control of reactive oxygen species may be responsible for many of the clinical manifestations of this disease. To the best of our knowledge, hepatic lesions of steatohepatitis have not previously been reported in A-T patients. The present study reports the case of a 22-year-old man diagnosed with A-T at the age of 6 years who was referred to our Digestive Disease Unit with a three-year history of hyperlipidemia and liver test alterations. Core liver biopsy showed similar lesions to those observed in nonalcoholic steatohepatitis. Immunohistochemical staining disclosed the absence of ATM protein in hepatocyte nuclei. We suggest that the liver injury may be mainly attributable to the oxidative stress associated with ATM protein deficiency, although other factors may have made a contribution. We propose the inclusion of A-T among the causes of nonalcoholic steatohepatitis, which may respond to antioxidant therapy.

## 1. Introduction

Ataxia-telangiectasia (A-T) is a rare autosomal recessive hereditary neurodegenerative and progressive disease caused by mutations in the ataxia-telangiectasia-mutated (ATM) gene that produce the absence or inactivation of ATM protein kinase. Clinical manifestations of A-T include early-onset neurological alterations (cerebellar ataxia caused by Purkinje and granule cell degeneration), late-onset oculocutaneous telangiectasias, early aging, sterility, hypersensitivity to ionizing radiation, immunodeficiency, and susceptibility to neoplasms [1, 2], especially leukemia, lymphomas, and breast cancer [3, 4]. Patients with A-T can also have impaired cellular and humoral immunity (IgA, IgE, or IgG2 immunodeficiency) and elevated serum alpha-fetoprotein (AFP), which can be useful for the diagnosis [4, 5].

ATM protein participates in double-strand-break repair mechanisms and can be activated by exogenous and endogen oxidative stress; ATM activation increases antioxidant levels and induces DNA oxidative damage repair [6]. Along with p53, ATM plays an important role in maintaining genomic integrity [5]. Many of the clinical alterations observed in A-T patients may be related to the dysfunctional control of reactive oxygen species (ROS) observed when ATM is deficient [1].

Nonalcoholic steatohepatitis (NASH) is a progressive form of nonalcoholic fatty liver disease histologically characterized by hepatocyte steatosis, ballooning (with or without Mallory-Denk body [MDB] formation), and lobular necroinflammatory lesions, which tend to be associated with pericellular and perisinusoidal fibrosis; many of these changes are localized in the acinar zone 3 [7, 8]. Although various factors may contribute to the development of these alterations, ROS-mediated oxidative stress is known to play a major role in their genesis [9, 10].

We present the first report of NASH lesions in an A-T patient who had shown alterations in liver tests over the previous two years. We discuss the pathogenic mechanisms that may be implicated in the genesis of liver damage in this disease.

## 2. Case Report and Results

A 22-year-old male, with a height of 160 cm and body mass index of 17.5, was referred to the Department of Digestive Diseases for elevated serum transaminases. The family history was not relevant. He was diagnosed with A-T in infancy, and cerebellar atrophy was revealed by magnetic resonance imaging at the age of six years. At the time of his referral, his progressive motor alteration had left him wheelchair bound. His history also included agammaglobulinemia (IgA) since childhood, requiring immunoglobulin substitution therapy; H1N1 influenza A virus infection; repeated respiratory infections; and recurrent herpetic keratitis, treated with valacyclovir for the previous 10 years.

Tests over the two years before his referral to the Digestive Disease Unit evidenced elevated serum AST, ALT, and GGT values and dyslipidemia; in the biopsy taken at his referral, the serum values were 204 U/L (N ≤ 37), 376 U/L (N ≤ 40), and 442 U/L (N ≤ 50), respectively. Serum TG (167 mg/dL, N ≤ 150) and LDL (139 mg/dL, N ≤ 130) levels were mildly elevated, HDL was normal, and he evidenced thrombocytosis (502 × 10^3^ *μ*L) and a very high AFP level (1202 ng/mL; N ≤ 10). Blood pressure, basal glucose, thyroid hormones, and alfa-1 antitrypsin values were normal; viral serology (HAV, HBV, HCV, HEV, CMV, HSV, EBV, VZV) and autoantibody (ANA, AMA, AML, LKM1, ATA) screening results were negative, and no iron or copper metabolism anomalies were detected. Ultrasound scan detected liver steatosis. A percutaneous liver biopsy was taken.

Liver biopsy was fixed in 10% neutral formalin and embedded in paraffin; 4 *μ*m sections were obtained and stained with hematoxylin-eosin, PAS-diastase, Gomori trichrome, Gordon-Sweet reticulin, and Prussian blue (Perls). Immunohistochemical techniques were also applied with an automated system (Lab Vision Autostainer 720) using primary antibodies against p62 protein (monoclonal antibody 3P62LCK, 1/500 dilution, BD transduction), which binds to MDBs, and against ATM protein (monoclonal antibody Y170, prediluted, Master Diagnóstica, Granada, Spain).

**Figure 1:**
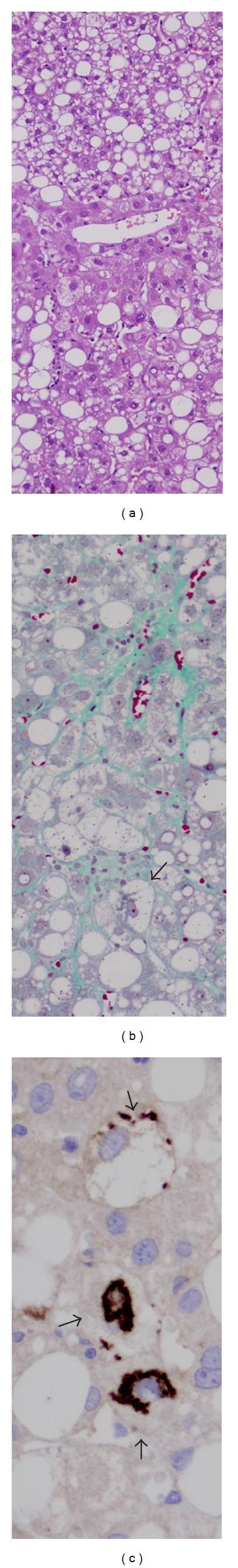
Histological changes in liver biopsy from patient with ataxia-telangiectasia. Hepatic lobule showing hepatocytes with macrovesicular and multivesicular steatosis (hematoxylin-eosin (a)) and perisinusoidal and pericellular fibrosis around ballooning hepatocytes, some containing Mallory-Denk bodies (arrow) (Gomori trichrome (b)), which are more evident with immunohistochemical staining for p62 protein, arrows (c) (original magnification: ×10, ×20, and ×40, resp.).

**Figure 2:**
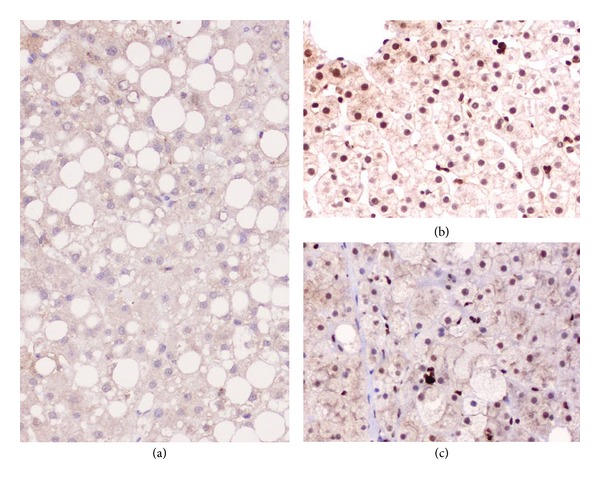
Immunohistochemical staining of liver biopsies with anti-ATM antibody. Absence of hepatocyte nuclear staining in the A-T patient (a). Nuclear immunostaining is observed in a normal liver (b) and in a liver biopsy specimen from an obese patient with nonalcoholic steatohepatitis (c) (immunoperoxidase, original magnification: ×20).

Histological examination of the liver biopsy revealed the characteristic features of steatohepatitis, that is, moderate macrovesicular and multivesicular steatosis (Figure 1(a)) and ballooning hepatocytes, some containing MDBs, in perivenular areas, along with pericellular and perisinusoidal fibrosis (Figure 1(b)). Some foci of lobular inflammation were also observed, but no fibrosis or inflammatory infiltration of portal tracts was detected; therefore, the lesions were classified as stage 1A. Immunohistochemical study with the anti-p62 antibody revealed the presence of MDBs in the cytoplasm of several ballooning hepatocytes localized in acinar zone 3 of numerous lobules (Figure 1(c)). Staining for ATM protein showed that expression of this protein was absent in the hepatocyte nuclei (Figure 2(a)), whereas it was found in normal liver and in liver specimens from patients with steatohepatitis of different etiologies (Figures 2(b) and 2(c)) (unpublished observation).

## 3. Discussion

A-T is a rare hereditary and recessive disease whose clinical manifestations are related to the presence of a defective or nonfunctional ATM protein due to ATM gene mutation [1, 5]. A-T is a clinically heterogeneous disease, and there are milder forms with a slower or later neurological progression [2] that may produce various neuropathological alterations [11].

ATM protein is a serine/threonine protein kinase, chief activator of the DNA damage response induced by DNA double-strand breaks after ionizing radiation and other insults, and it may be activated by oxidative stress [1, 5, 6]. ATM protein is involved in several cellular biological processes such as neural and immune system homeostasis, cell cycle checkpoints, genomic stability and insulin signaling, among others, which are controlled by signaling pathways triggered after ATM activation [11–13]. ATM protein is distributed in the cytoplasm and, mainly, in the nucleus of the various types of cells in which it is abundant [13].

Many of the alterations observed in A-T patients may be related to the dysfunctional control of ROS, given that cells lacking ATM exhibit high ROS concentrations and hypersensitivity to oxidative stress-inducing agents [1, 6]. Chronic activation of stress response pathways has been observed in tissues with pathological changes, such as the cerebellum, and other areas of the central nervous system may become affected if the life of the patient is prolonged [1, 10].

Serum AFP level was elevated in our patient, as also reported in more than 95% of A-T patients [5], and this elevation may be of value in the differential diagnosis between A-T and other A-T-like diseases. The increased serum AFP in A-T is not related to hepatic injury and is of uncertain origin, although a relationship with cerebellar Purkinje cell degeneration has been proposed, based on the elevated AFP levels observed in various diseases characterized by neural tube defects [4]. High AFP levels have also been described in diseases produced by AFP gene mutation, including hereditary persistence of alpha-fetoprotein [14]; inflammatory liver diseases, usually associated with malignant transformation; and germinal cell neoplasms.

NASH, a potentially aggressive and progressive form of nonalcoholic fatty liver disease, displays similar histological lesions to those in alcoholic hepatitis, with a distribution in the acinar zone 3 of hepatic lobules and a variable severity [7], although MDBs and fibrosis are less pronounced in NASH, as in the present case. The origin of these lesions is multifactorial, although an important role is played by ROS-induced oxidative damage [9], which produces injury in several cell components, including protein and DNA damage and membrane lipid peroxidation. The characteristic histologic lesions of NASH (ballooning, MDBs, necrosis/apoptosis of hepatocytes, inflammation, and fibrosis) represent the morphological expression of oxidative stress [8, 15].

Steatosis is the first event (“hit”) in the development of steatohepatitis [16]. Macrovesicular steatosis can be induced if mild and prolonged alteration of mitochondrial *β*-oxidation occurs [17], which may explain the onset of liver injury in this patient, along with other factors (e.g., dyslipidemia).

Although the most common cause of nonalcoholic fatty liver disease is the metabolic syndrome, it has also been related to other causes, including nutritional and metabolic status, drugs, genetic factors, and infectious agents, among others [8]. However, A-T has never been cited as a possible etiology.

Patients with A-T are reported to be at increased risk of diabetes mellitus type 2 (DM-2), although their short life expectancy and the typically late onset of DM-2 means that its associated complications are not usually observed in these patients [12]. DM-2 may be associated with metabolic syndrome and nonalcoholic fatty liver disease. Basal glucose levels were normal in the patient in all determinations carried out during the followup; although the patient showed elevated TG and HDL when the biopsy was taken, these values are normal at present after dietetic modification and statin treatment. No other parameters of metabolic syndrome were altered.

NASH and A-T share the same pathogenic mechanism of ROS generation. ROS may contribute to the mitochondrial dysfunction that plays a role in the development of lesions in NASH, which has been considered a mitochondrial disease [15]. In turn, excessive ROS production has been attributed to mitochondrial dysfunction in other diseases, including neurodegenerative conditions [13], and may be induced by the morphofunctional mitochondrial changes in A-T patients.

The development of steatohepatitis in our patient may be the result of oxidative stress due to inactive ATM protein, although other possible contributory factors include dyslipidemia, physical inactivity, and pharmacological treatments, which can induce further ROS generation and/or antioxidant depletion of hepatocytes.

The relatively long survival of the patient, probably attributable to his less severe form of A-T, may be responsible for the development of the hepatic lesions. We propose A-T as a candidate for inclusion among the causes of NASH. Further studies of larger series of patients with A-T are required to confirm our observations, although this is a challenging task given the low incidence of this disease (1 per 40,000–100,000 newborns [5]).

In summary, A-T patients with a relatively long survival may develop hepatic lesions similar to those in nonalcoholic steatohepatitis, and A-T should therefore be considered as a possible cause of NASH. Oxidative stress due to the functional deficiency of ATM protein is involved in the pathogenesis of hepatic lesions in this patient, which may therefore be susceptible to antioxidant therapy. We propose that NASH may be considered as a late phenotypic manifestation of A-T.

## Abbreviations

A-T: Ataxia-telangiectasia
ATM: Ataxia telangiectasia mutated
AFP: Alpha-fetoprotein
ROS: Reactive oxygen species
NASH: Nonalcoholic steatohepatitis
MDB: Mallory-Denk body
ALT: Alanine aminotransferase
AST: Aspartate aminotransferase
GGT: Gamma-glutamyltransferase
N: Normal
TG: Triglycerides
LDL: Low-density lipoproteins
HDL: High-density lipoproteins
HAV: Hepatitis A virus
HBV: Hepatitis B virus
HCV: Hepatitis C virus
HEV: Hepatitis E virus
Ab: Antibody
CMV: Cytomegalovirus
EBV: Epstein-Barr virus
HSV: Herpes simplex virus
VZV: Varicella zoster virus
ANA: Anti-nuclear Abs
AMA: Anti-mitochondrial Abs
SML: Smooth-muscle Abs
LKM1: Liver/kidney microsomal Abs
ATA: Anti-transglutaminase Abs
DM-2: Diabetes mellitus-type 2.

## References

[1] Barzilai A, Rotman G, Shiloh Y (2002). ATM deficiency and oxidative stress: a new dimension of defective response to DNA damage. *DNA Repair*.

[2] Taylor AMR, Byrd PJ (2005). Molecular pathology of ataxia telangiectasia. *Journal of Clinical Pathology*.

[3] Boultwood J (2001). Ataxia telangiectasia gene mutations in leukaemia and lymphoma. *Journal of Clinical Pathology*.

[4] Ball LG, Xiao W (2005). Molecular basis of ataxia telangiectasia and related diseases. *Acta Pharmacologica Sinica*.

[5] Mavrou A, Tsangaris GT, Roma E, Kolialexi A (2008). The ATM gene and ataxia telangiectasia. *Anticancer Research*.

[6] Guo Z, Deshpande R, Paull TT (2010). ATM activation in the presence of oxidative stress. *Cell Cycle*.

[7] Brunt EM (2001). Nonalcoholic steatohepatitis: definition and pathology. *Seminars in Liver Disease*.

[8] Angulo P (2002). Medical progress: nonalcoholic fatty liver disease. *New England Journal of Medicine*.

[9] Parola M, Robino G (2001). Oxidative stress-related molecules and liver fibrosis. *Journal of Hepatology*.

[10] Verhagen MMM, Martin J-J, van Deuren M (2012). Neuropathology in classical and variant ataxia-telangiectasia. *Neuropathology*.

[11] McKinnon PJ (2012). ATM and the molecular pathogenesis of ataxia telangiectasia. *Annual Review of Pathology: Mechanisms of Disease*.

[12] Ditch S, Paull TT (2012). The ATM protein kinase and cellular redox signaling: beyond the DNA damage response. *Trends in Biochemical Sciences*.

[13] Ambrose M, Gatti RA (2013). Pathogenesis of ataxia-telangiectasia: the next generation of ATM functions. *Blood*.

[14] Blesa JR, Giner-Durán R, Vidal J (2003). Report of hereditary persistence of α-fetoprotein in a Spanish family: molecular basis and clinical concerns. *Journal of Hepatology*.

[15] Pessayre D, Fromenty B (2005). NASH: a mitochondrial disease. *Journal of Hepatology*.

[16] Day CP, James OFW (1998). Steatohepatitis: a tale of two “Hits”?. *Gastroenterology*.

[17] Labbe G, Pessayre D, Fromenty B (2008). Drug-induced liver injury through mitochondrial dysfunction: mechanisms and detection during preclinical safety studies. *Fundamental and Clinical Pharmacology*.

